# A review on individual and multistakeholder fairness in tourism recommender systems

**DOI:** 10.3389/fdata.2023.1168692

**Published:** 2023-05-10

**Authors:** Ashmi Banerjee, Paromita Banik, Wolfgang Wörndl

**Affiliations:** Department of Computer Engineering, TUM School of CIT, Technical University of Munich, Munich, Germany

**Keywords:** tourism recommender systems, travel, information retrieval, multistakeholder recommendations, fairness

## Abstract

The growing use of Recommender Systems (RS) across various industries, including e-commerce, social media, news, travel, and tourism, has prompted researchers to examine these systems for any biases or fairness concerns. Fairness in RS is a multi-faceted concept ensuring fair outcomes for all stakeholders involved in the recommendation process, and its definition can vary based on the context and domain. This paper highlights the importance of evaluating RS from multiple stakeholders' perspectives, specifically focusing on Tourism Recommender Systems (TRS). Stakeholders in TRS are categorized based on their main fairness criteria, and the paper reviews state-of-the-art research on TRS fairness from various viewpoints. It also outlines the challenges, potential solutions, and research gaps in developing fair TRS. The paper concludes that designing fair TRS is a multi-dimensional process that requires consideration not only of the other stakeholders but also of the environmental impact and effects of overtourism and undertourism.

## 1. Introduction

Recommender Systems (RS) are utilized across various domains to provide personalized access to information and help users navigate through vast amounts of content. In e-commerce, media, entertainment, and other industries, they improve the user experience, increase engagement, boost sales, and drive revenue. By enhancing the discoverability of relevant items, RS ultimately lead to greater satisfaction and loyalty among users (Ricci et al., 2020).

In the past, evaluating the effectiveness of recommender systems was mainly based on their ability to cater to the needs and preferences of end users. This approach makes sense as users would not use the systems if it does not meet their interests. However, RS have seen a tremendous gain in popularity and have now impact beyond the users they were initially designed for. Therefore, it is important to note that in many cases, the end user is not the only stakeholder impacted by the recommendations. Other users, product providers, and the system's goals should also be taken into account. This has led to the inclusion of objectives like fairness and balance in the evaluation process, even if they may not align with individual preferences. Focusing solely on the end user limits the ability to consider the concerns of the other stakeholders in the design and algorithm of recommender systems (Abdollahpouri et al., 2020). Hence, a fair recommender system is evaluated from various stakeholders' perspectives, making it a complex and multi-faceted process (Burke, 2017).

The widespread adoption of RSin the travel industry has made trip planning easier for travelers by offering personalized recommendations for destinations, accommodations, activities, etc. (Isinkaye et al., 2015). Tourism Recommender Systems (TRS) stand out from other RS domains due to their susceptibility to dynamic factors that are subject to frequent changes. For instance, changes in seasonality or travel regulations can have a significant impact on travel plans (Balakrishnan and Wörndl, 2021). Furthermore, it also involves capacity-limited items, including airline seats, hotel rooms, and tickets to events, further aggravating the complexity of the domain (Abdollahpouri and Burke, 2021).

In the realm of tourism, where recommendations can greatly impact not only the end user but also the local community and the environment, it becomes even more crucial to evaluate recommender systems from multiple perspectives and strive for fairness in their recommendations. The travel and tourism domain is complex, encompassing various stakeholders beyond just the traveler, such as transportation providers, host destinations, and information platforms, each with their own needs and goals (Abdollahpouri et al., 2020). Additionally, while constructing a fair TRS, it is important to take into account the environmental impact of tourism. Tourism and the environment are intertwined in a complex relationship that includes activities that can have both negative and positive impacts. On one hand, tourism can contribute to environmental protection and conservation, raise awareness of environmental values, and provide funding for natural areas. On the other hand, it can also have adverse effects such as contributing to climate change, depleting natural resources, causing overtourism or undertourism, etc. (Camarda and Grassini, 2003; Gössling, 2017).

A well-designed TRS can be particularly beneficial in controlling the influx of tourists to a region. Such control is essential in addressing two related problems that have become increasingly prevalent in recent years: overtourism and undertourism. The growth of low-cost aviation, cheap transportation, social media popularity, and home-sharing platforms like Airbnb^1^ have led to a surge in visitors to popular destinations, resulting in overtourism. At the same time, there are under-explored destinations that suffer from undertourism due to a lack of infrastructure, publicity, and accessibility (Gowreesunkar and Vo Thanh, 2020). Both over and undertourism have several negative consequences. Overtourism endangers the preservation of the city's historic center and has negative consequences for the environment, residents, and tourists' experiences, making it challenging to find reasonably priced housing in these cities (Dastgerdi and De Luca, 2023). Cities in Europe such as Venice, Barcelona, Rome, and Dubrovnik are grappling with the effects of overtourism (Dodds and Butler, 2019). A lack of tourists on the other hand can have adverse effects as well as experienced during the Covid-19 pandemic outbreak. The pandemic had a profound impact on the tourism industry, causing severe disruptions to the tourism and hotel industries (Hao et al., 2020; Gaĺı Espelt, 2022).

To help mitigate these and other problems TRS should be designed to take into account the interests of all stakeholders, advocate for sustainable tourism practices, and encourage responsible tourism while providing recommendations to users. To this end, our work makes the following three contributions:

- We highlight the main fairness criteria and categorize stakeholders based on the ones that apply to them.
- We review state-of-the-art research on TRS fairness from multiple stakeholder perspectives.
- Finally, we outline the challenges, potential solutions, and research gaps to lay the foundation for future research in developing fair TRS.

The paper is structured as follows: we begin with an overview of fairness in RSand the stakeholders involved in TRS in Section 2.1 and Section 2.2. Next, in Section 2.3, we explain our methodology for identifying relevant papers for our survey and provide some statistical information on the papers reviewed. We then delve into the concept of individual or intra-stakeholder fairness in Section 3 and examine works that focus on multiple stakeholders simultaneously in Section 4. Finally, in Section 5, we conclude the paper by discussing the challenges encountered by TRSand potential solutions to address them.

## 2. Terminology

### 2.1. Fairness in RS

In an era, where data drives decisions, it is crucial to examine if algorithms may discriminate based on gender, ethnicity, or other protected attributes. Multiple studies have investigated fairness in decision-making systems based on Machine Learning methods (Pedreshi et al., 2008; Zemel et al., 2013; Hardt et al., 2016; Zafar et al., 2017; Speicher et al., 2018), and Information Retrieval (Castillo et al., 2017; Yang and Stoyanovich, 2017; Biega et al., 2018; Celis et al., 2018; Singh and Joachims, 2018).

A multitude of fairness notions has been studied to ensure that algorithmic decisions are fair. They can be divided into *individual* and *group fairness* notions. Group fairness ensures fair treatment of similar subjects within the different groups based on protected attributes such as race or gender (Masthoff and Delić, 2012). Individual fairness assesses whether individuals are treated fairly by ensuring that similar subjects receive similar decision outcomes (Dwork et al., 2012).

The concept of fairness applies to RStoo. RSoffer personalized access to a vast amount of content across domains like e-commerce, social media, news, travel, and more, finding relevant information and avoiding information overload (Abdollahpouri et al., 2020). They are usually evaluated for recommendation accuracy, i.e., their ability to provide a list of items that meet the user's needs. However, increased awareness of fairness and bias issues in algorithmic decision-making (Romei and Ruggieri, 2014) have led researchers to focus on fairness aspects in RSevaluations (Kamishima et al., 2013; Burke, 2017; Serbos et al., 2017; Xiao et al., 2017; Yao and Huang, 2017; Burke et al., 2018; Liu and Burke, 2018; Steck, 2018; Abdollahpouri et al., 2020).

While the notions of individual and group fairness can be applied to RSas well, fair machine learning differs from fairness in RSthrough the multi-sided nature of the latter. Fairness in recommendation systems is often a multi-sided concept that takes into account the needs and perspectives of multiple stakeholders (Burke, 2017). In other words, there may be multiple fairness-related criteria at play in determining fair outcomes and these outcomes cannot be evaluated based solely on the results for one side of a transaction. In RS, a *stakeholder* is any group or individual that can be affected by or can affect the delivery of recommendations (Abdollahpouri et al., 2020). Therefore, a *multistakeholder* RSshould serve the goals of all stakeholders involved. However, in practice, this is often not the case, which is attributed to the existence of different biases in RS.

RS can exhibit the following three types of common biases: *popularity, exposure, ranking*, or *position bias*. *Popularity bias* is a major fairness concern in recommendation systems. It refers to the tendency of the system to recommend items that are often popular among users, regardless of the individual preferences of a particular user (Abdollahpouri et al., 2019a). This can often result in less popular items being disfavored, leading to unfair recommendations in terms of the exposure given to different items of varying popularity, known as *exposure bias* (Abdollahpouri and Mansoury, 2020). In recommender systems, rankings of items play an integral role in the decision-making process. As ranking positions influence the amount of attention received by the ranked items, biases in rankings can lead to the unfair distribution of resources and opportunities (Biega et al., 2018). This type of bias is known as the *ranking bias* or *position bias* in the literature.

As pointed out by Buet-Golfouse and Utyagulov (2022), fairness definitions often vary based on domains and context. To study fairness in multistakeholder recommender systems, it is important to identify the stakeholders who should receive fair treatment, quantify any harms that may occur, and analyze metrics for measuring and minimizing these harms (Ekstrand et al., 2020). This process of defining an objective function involves taking a concern (in this case, reducing representational harm) and translating it into a specific framework and metric (Ekstrand et al., 2020). The resulting metric should also measure the usefulness i.e. the utility of the recommendations for the user. In our work, we conceptualize the *utility of a recommendation result for a multistakeholder system as its usefulness for each stakeholder*.

However, it is important to note that this process of defining a metric is inherently limited and may result in trade-offs. These limitations and trade-offs do not necessarily render the fairness construct invalid. All fairness constructs come with their limitations and trade-offs and there is no universally accepted definition of fairness (Narayanan, 2018; Ekstrand et al., 2020). In our work, *a multistakeholder recommender system is considered to be fair if it minimizes any bias or circumstance that may result in disfavored outcomes for each stakeholder*. This implies that a fair multistakeholder RS may have to consider trade-offs in the respective stakeholder concerns.

### 2.2. Stakeholders in TRS

In the tourism industry, the traveler is not the only stakeholder involved. Every service that is part of the traveler's journey, including transportation providers, host destinations, and information platforms, also has a stake in the industry (Abdollahpouri et al., 2020). Optimizing recommendations for the consumers' experience can often align with and benefit the goals of the providers, such as increased sales or higher engagement. However, there may also be situations where achieving the goals of one stakeholder may come at the expense of another stakeholder's goals, creating potential trade-offs (Jannach and Bauer, 2020).

Following the classification of common stakeholders encountered in a generic multistakeholder recommender system we generalize the type of stakeholders encountered in common touristic recommendation scenarios into the following classes. Our categorization is inspired by the work of Balakrishnan and Wörndl (2021).

- *Consumers*: the end users who receive or want to receive recommendations to plan their trips, such as tourists, business travelers, airline passengers, etc.
- *Item Providers*: the entities that provide the consumers with the recommended facility for their trips, such as hotels, resorts, rentals, amusement parks, airlines, tour operators, and vacation companies.
- *Platform*: the recommender system itself, such as flight booking platforms, vacation recommenders, city information systems, travel sites, e-commerce sites, hotel platforms, and similar systems.
- *Society*: it represents the environment and entities or groups that are affected by the tourism activity but are not directly part of the TRS. This can include the local environment, city authorities, municipal councils, local businesses, and Destination Management Organizations (DMOs).

Although the aforementioned stakeholder categorization seems plausible, stakeholder relationships, in reality, can be more complex. For instance, in the context of tourism's value chain, the providers of final services (e.g., hotels), travel companies, online travel platforms, and even travel agencies can be further subdivided despite being grouped as item providers. This grouping may create a false impression that they all share the same interests, which is not the case and could impact fairness for these different groups. However, this is the most logical and simplified approach to structuring the stakeholders based on earlier works by Abdollahpouri et al. (2020), Jannach and Bauer (2020). On the other hand, the inclusion of society as a stakeholder in tourism by Jannach and Bauer (2020); Balakrishnan and Wörndl (2021) is a novel and appropriate perspective. This viewpoint adds an interesting dimension to the functioning of multistakeholder tourism, emphasizing the crucial issue of reducing the environmental impact caused by tourist activities.

**Figure 1:**
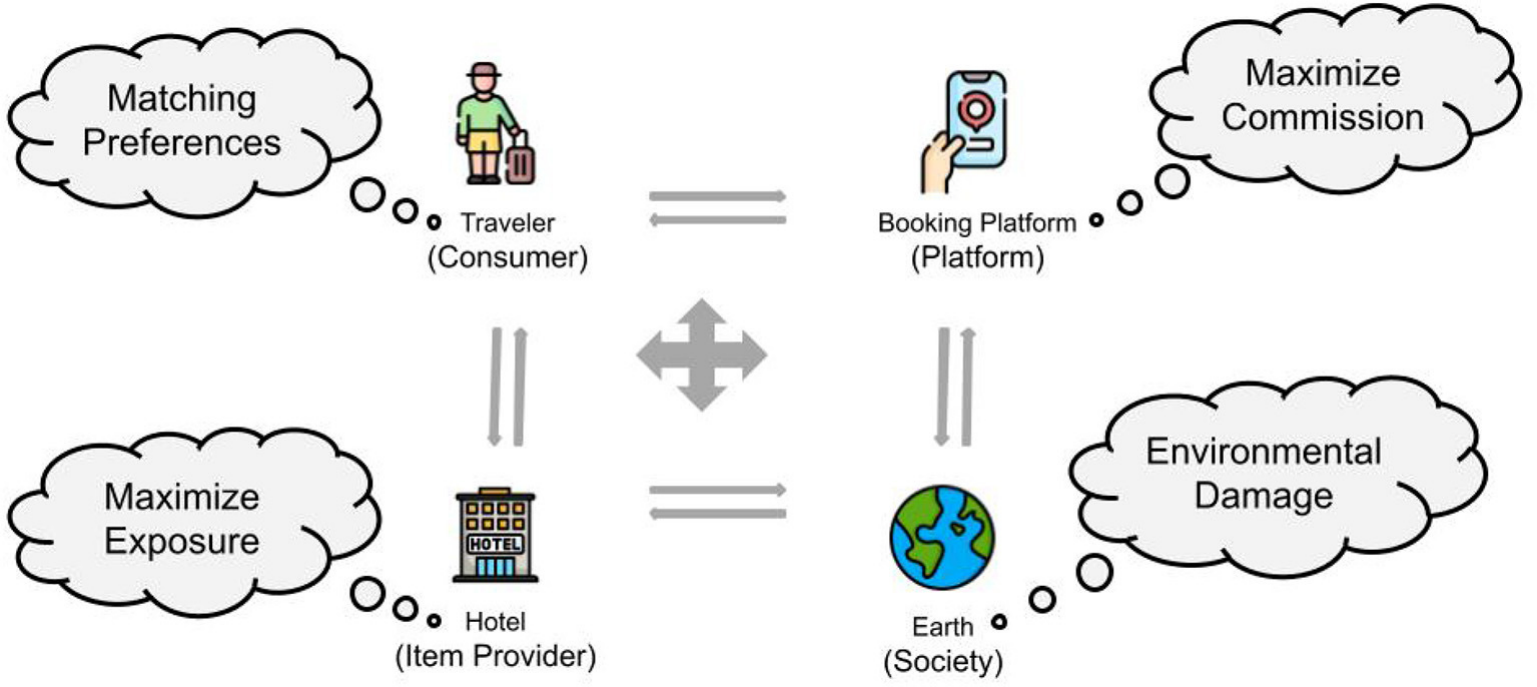
The multistakeholder environment in a hotel booking scenario adapted from Abdollahpouri and Burke (2019).

To understand the stakeholder interplay, let us consider the example of a hotel booking scenario on a platform like Booking.com^2^ in Figure 1. Here, we can observe all four major stakeholders as shown: (1) end users or travelers who are searching for accommodation in the city during the specified period, (2) the hotels that are being recommended, (3) the booking platform itself that is providing the recommendations for hotels and 4) Society i.e. city authorities, municipal councils, and DMOs who must ensure that the city is not over-crowded and the environment is not compromised.

Travelers want to find hotels that match their preferences, hotels want fair exposure to attract guests, and booking platforms want to maximize the commission received from the hotels and maintain long-term relationships with both users and hotel providers. All stakeholders are dependent on each other for their economic well-being, and therefore the booking platform must take all stakeholders' preferences into account when generating recommendations. Additionally, society plays a key role in ensuring minimal environmental impact and avoiding overcrowding in the city. As a responsible platform, Booking.com should take into consideration the concerns of indirectly affected actors from society. This example reinstates the domain of tourism as a prime use case for studying multistakeholder recommender systems, where different stakeholders interact with one another directly or indirectly. As the example shows, these stakeholders are often interdependent for their existence. Furthermore, in certain situations, stakeholders may play multiple roles and should be considered separately as distinct entities, as discussed by Balakrishnan and Wörndl (2021).

We use analogous terminology as Abdollahpouri and Burke (2019) to demonstrate the close connection between multistakeholder recommendation and multi-sided fairness. We categorize fairness into four groups— *C-Fairness*, which focuses on consumers and encompasses *individual* and *group discrimination*; *I-Fairness*, which targets item providers and deals with *popularity bias* and *exposure bias*; *P-Fairness*, which concentrates on platforms and addresses *ranking bias*; and *S-Fairness*, which takes into account the impact on society through *sustainability*. These groups provide an intra-stakeholder perspective on fairness along with their respective key fairness criteria. Each group has been further studied with different fairness attributes as summarized in Table 1. The overlapping stakeholder scenarios have been addressed in Section 4.

**Table 1:** Table summarizing related works in fairness from different stakeholder perspectives, that directly or indirectly contribute to Tourism Recommender Systems.

**Fairness type**	**Stakeholder focus**	**Main fairness criteria**	**References**
C-Fairness	Consumers/ End-Users	Individual Discrimination	Edelman et al. (2017); Serbos et al. (2017)
			Herzog and Wörndl (2019); Jaeger and Sleegers (2020)
			Zhang et al. (2022)
		Group Discrimination	Delic et al. (2018); Mansoury et al. (2019)
			Rahmani et al. (2022a)
I-Fairness	Item-providers/ Producers	Popularity Bias	Jannach et al. (2015); Fu et al. (2021)
			Pala (2021); Wei et al. (2021)
			Lin et al. (2022); Tacli et al. (2022)
			Zhou et al. (2020); Yalcin and Bilge (2022)
		Exposure Bias	Abdollahpouri and Mansoury (2020); Banerjee et al. (2020)
			Khenissi and Nasraoui (2020); Gupta et al. (2021b)
			Yang et al. (2021)
P-Fairness	Platforms/ Systems	Ranking Bias	Biega et al. (2018); Grbovic and Cheng (2018); Lahoti et al. (2019)
			Gunawardena and Sarathchandra (2020); Kokkodis and Lappas (2020)
			Li (2020); Mavridis et al. (2020)
			Zhu et al. (2020); Gupta et al. (2021a)
			Kangas et al. (2021); Gupta et al. (2022)
S-Fairness	Society	Sustainability	Patro et al. (2020b); Pachot et al. (2021)
			Merinov et al. (2022)

### 2.3. Research methodology

The following section outlines our approach to identifying pertinent papers for the survey. We will then provide a brief overview of how our survey builds upon previous research in this field.

Firstly, we developed a methodology to identify relevant papers for our survey. We began by using predefined search terms and explicit inclusion and exclusion criteria to query the Google Scholar web search engine. Additionally, we employed a snowballing technique and relied on researcher experience to identify any relevant papers that were not captured by our initial search.

To ensure that we covered a broad range of works in an emerging field with inconsistent terminology, we used the following keywords: *tourism, fairness, multistakeholder*, and *recommender systems*. Later, to identify more studies specific to the tourism industry, we expanded our search terms to include relevant terms to tourism platforms such as *Airbnb, TripAdvisor, Yelp*, and Booking.com.

Finally, we manually reviewed the resulting papers to determine if they met the following criteria for inclusion in our survey:

- It had to include at least one fairness criterion or bias in RS as identified in Table 1.
- It has to be within tourism or a comparable domain.
- It has to be about RS, ranking, or information retrieval.
- It has to be published in the last decade except for two papers that were included due to their significant conceptual contributions to the field of ranking, rather than their specific use cases.

**Figure 2:**
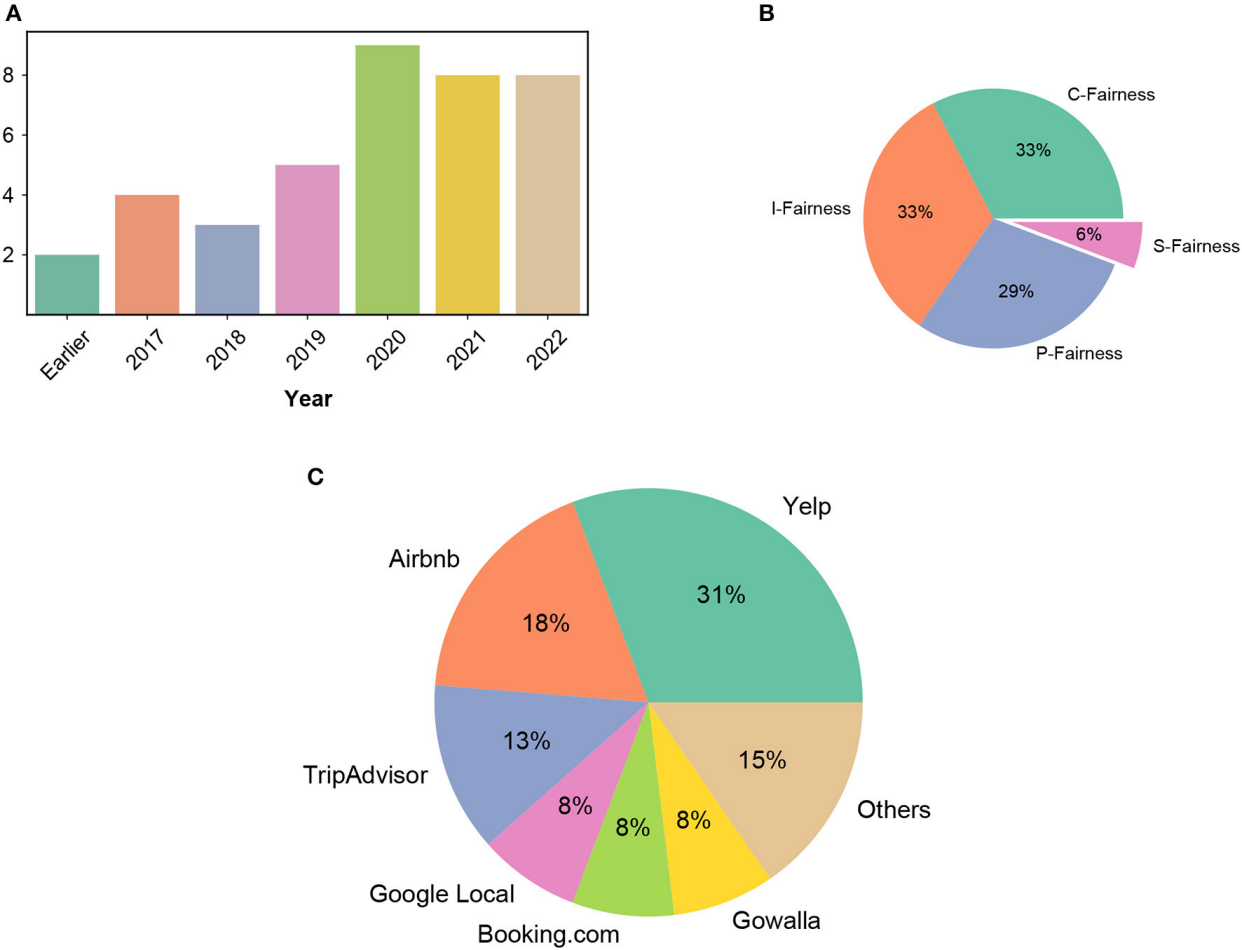
**(A)** The number of papers on fairness in TRS published per year. The total number of papers amounts to 66. **(B)** Percentage of papers with each stakeholder focus in TRS. **(C)** Percentage of papers analyzing each dataset in TRS.

In this process, we identified a total of 66 papers, which we divided into TRS and non-TRS categories based on the domain in which they focussed. Figure 2A illustrates the number of papers on fairness in TRS published per year and from the visualization, it is evident that fairness in TRS is a relatively nascent field, with the most recent research dating back to 2014.

The resulting papers for TRS were systematically analyzed based on four distinct aspects: the specific fairness criteria or bias being addressed, solutions proposed, results evaluated, and datasets analyzed. According to our analysis presented in Figure 2B, previous research has primarily focused on fairness to consumers, item providers, and platforms, with very little attention given to fairness to society as a stakeholder. We further discuss this in detail in Section 3. As depicted in Figure 2C, various datasets from the travel and tourism domain were utilized in the studies analyzed.

It is worth noting that fairness in RS has been extensively surveyed in literature in recent years, as shown by authors such as Deldjoo et al. (2022) and Wang et al. (2022), who provide in-depth reviews of related concepts and work on the particular topic. However, our study aimed to provide a comprehensive understanding of fairness from multiple stakeholders' perspectives within TRS, with a specific focus on society. As a result, our survey differs from previous surveys in that our objective was to investigate and identify research gaps in the current state of existing research for TRS.

## 3. Individual stakeholder fairness in TRS

In this section, we discuss related papers including fairness concerns that were gathered according to the explained methodology in Section 2.3. Each subsection focuses on stakeholder fairness with respect to the primary fairness criteria presented in Table 1, and it examines relevant literature falling under those criteria. The approaches outlined in Table 1 primarily address one primary stakeholder and fairness criterion, but they can be applied, to some extent, to other stakeholders as well. For instance, while popularity bias and exposure bias can have an impact on the platform itself, their primary effects are on the providers of the items. Hence, for the sake of simplicity, we have chosen to address the primary fairness criteria to a single stakeholder.

### 3.1. Consumer fairness: individual and group discriminations

Consumer Fairness (C-Fairness) refers to the need for a recommender system to consider the different effects of its recommendations on the protected or sensitive attributes of its users, such as age, gender, and nationality. It also encompasses any fairness concerns the system may have concerning its users (Sonboli et al., 2021).

C-Fairness can appear on an individual as well as a group level. Individual Fairness refers to treating similar individuals in a similar way (Dwork et al., 2012). In a group recommender system, this means considering the preferences of all group members fairly and not ignoring any individual's preferences (Masthoff and Delić, 2012).

Despite a significant amount of research on C-Fairness in other application domains, such as music recommendations (Dinnissen and Bauer, 2022), the field of travel and tourism has seen relatively little analysis of this topic. In the tourism industry, researchers often examined the effect of a user's gender and the business category on various outcomes (Mansoury et al., 2019). For example, a study on Airbnb revealed that hosts who had never hosted African American guests were less likely to accept guests with African American names compared to those with White names (Edelman et al., 2017). Another analysis showed that non-White hosts charge 2.5–3% lower prices for similar listings, while Black and Asian hosts charge approximately 5–7% and 4–6% less respectively (Jaeger and Sleegers, 2020). It was also found that discrimination between hosts and guests on Airbnb is reciprocal, with specific topics in reviews and self-descriptions significantly associated with discrimination (Zhang et al., 2022).

Group recommender processes in the tourism industry have been explored by Delic et al. (2018). The study aimed to observe the evolution of user preferences and interactions as a group during a tourism decision-making task. The authors also provided a comprehensive description of the study's data collection procedure, which can be utilized for further analysis to gain a deeper understanding of group decision-making processes.

Rahmani et al. (2022a) explored the impact of adding contextual information (such as geographic, temporal, social, and categorical details) on the quality of point-of-interest recommendations. They focused on four aspects: accuracy, novelty, diversity, coverage, fairness, and interpretability. The authors developed a linear regression approach for combining contextual information from different sources and applied it to two datasets (Gowalla^3^ and Yelp Challenge^4^) to assess the fairness of recommendations for both active and inactive users and popular and less popular items. Their results suggest that context-aware recommendation methods tend to be fairer to both users and item providers compared to traditional collaborative filtering methods.

While most of the aforementioned studies aim at being fair to a group of users, the work by Serbos et al. (2017) propose envy-free tour package recommendations for travel booking sites to ensure each individual is satisfied with their recommendation, demonstrating their findings on the Yelp Challenge dataset. An analogous concept was covered by Herzog and Wörndl (2019), where they focused on individual fairness in user groups and addressed the recommendations of points of interest (POIs) based on group preferences often tend to be unfair for some group members. The authors proposed a distributed Group Recommender System (GRS) that aggregates all group members' individual preferences fairly with the option to share one display for all members to openly discuss their preferences. Their study results showed that the approach could deliver fairer recommendations to groups with close relationships between members as they felt more comfortable specifying travel preferences as a group. Whereas groups with looser connections preferred to use separate devices to specify their preferences individually and to leave the preference aggregation to the GRS.

### 3.2. Item-provider fairness: popularity bias and exposure bias

The item providers are the entities that offer or support the recommended items. A recommender system that has an item provider fairness (I-Fairness) requirement should treat these providers of items in an equitable manner (Abdollahpouri and Burke, 2019; Abdollahpouri et al., 2020). Ensuring I-Fairness is particularly important in multistakeholder systems, as not recommending an item of quality can lead to economic hardship for the item provider and can also negatively impact market diversity by allowing certain providers to dominate (Banerjee et al., 2020). This section centers on the unfair treatment of item providers resulting from popularity bias and exposure bias.

In the context of recommender systems, an item's likelihood of being recommended to a user is not only based on the user's preferences but also on the item's popularity and visibility on the platform. Popularity bias is a common data bias that affects recommender systems, causing them to favor more popular items over less popular ones (Belloǵın et al., 2017). This can lead to a lack of representation and fairness for less popular items or items that are only popular among small groups of users (Park and Tuzhilin, 2008). This bias can also be seen as unjust to the providers of less popular or new items as few users rate them (Abdollahpouri et al., 2019a). Furthermore, a market that is dominated by popularity bias will not allow room for the exploration of new and obscure products and will be limited by a small number of well-known item providers leading to a lack of diversity, stifling innovation and creativity, ultimately limiting the market (Abdollahpouri et al., 2019a).

Various provider-side bias mitigation strategies have been suggested by other researchers. These include statistical parity (Yang and Stoyanovich, 2017), balanced neighborhoods (Burke et al., 2018), statistical independence (Kamishima et al., 2018), pairwise comparison (Beutel et al., 2019), data re-sampling (Ekstrand et al., 2018; Rastegarpanah et al., 2019; Boratto et al., 2021), etc. The study of popularity biases from the item providers' perspective remains a widely researched topic not only in tourism but also in other domains (Kamishima et al., 2014; Abdollahpouri et al., 2017; Abdollahpouri, 2019).

Lately, there has been a growing focus on analyzing popularity biases in TRS. The study by Jannach et al. (2015) using a hotel proprietary dataset from HRS.com^5^ found that popular recommendation techniques prioritize a small portion of items or top sellers, and have limited accuracy. The popularity bias in Yelp data was analyzed by Zhou et al. (2020). They concluded that models relying solely on positive reactions such as purchases or clicks result in less personalized recommendations and heightened popularity bias. To overcome this, they suggest incorporating implicit feedback and user-generated reviews, which provide a wealth of preference information for each user. The use of user-generated reviews was also explored by Pala (2021) using TripAdvisor data to compare top-ranked and least-ranked hotels. They found little difference in online review sentiment for both types of hotels, indicating that popularity does not solely reflect quality (Ciampaglia et al., 2018). The cause-effect relationship of popularity bias was addressed by Wei et al. (2021), where they estimated the direct effect of item properties on the ranking score, and then removed it to eliminate popularity bias. Their strategy was proven effective through extensive experiments on multiple real-world recommendation datasets, including Yelp and Gowalla.

Debiasing frameworks for addressing popularity bias in Conversational Recommender Systems (CRS) have been proposed by Fu et al. (2021) and Lin et al. (2022). through various debiasing frameworks. The former introduced metrics for quantifying popularity bias in CRSs, along with a debiasing framework, while the latter presented a framework that balances recommendation performance and item popularity in the CRS environment by combining dialogue context and historical user information. Their experiments on the Yelp dataset demonstrated a successful balance between the effectiveness of recommendations and the popularity of the items in the conversational recommendation system setting.

Popularity bias can often result in less popular items being disfavored, leading to unfair recommendations in terms of the exposure given to different items of varying popularity, known as exposure bias (Abdollahpouri and Mansoury, 2020). They propose metrics to quantify exposure bias from the perspective of the users and the providers by evaluating their research on Last. FM^6^ and MovieLens^7^ datasets. They show that when the recommendations are calibrated for the users in terms of popularity it will also benefit the providers by providing them with the exposure that they deserve, further reinforcing the idea that RS should be evaluated from multistakeholder viewpoints. Even though their work is based on the Last.Fm and MovieLens data, it can be translated into the travel domain such as for destinations/POIs recommendations displayed on different platforms. The studies by Tacli et al. (2022) and Yalcin and Bilge (2022) similarly address popularity bias in Yelp data by analyzing users' preferences for popular items. Tacli et al. (2022) suggest evaluating users' actual tendencies toward item popularity to provide more accurate individual recommendations.

The work by Banerjee et al. (2020) quantify exposure bias arising from popularity and position bias in the case of location-based searches. Their experimental evaluation of multiple real-world datasets from Google, Yelp, and Booking.com reveal the existence of exposure disparity on these platforms. Exposure bias has been addressed from a causality perspective by Yang et al. (2021). They argue that a combination of deep learning techniques along with causal inference is an effective method to mitigate exposure bias in RS. The studies by Khenissi and Nasraoui (2020) and Gupta et al. (2021b) also propose novel methodologies to model and mitigate exposure bias. Even though their work is demonstrated on the MovieLens dataset and for citation link recommendations respectively, the concept and methodology can be translated to the domain of tourism as potential strategies to minimize exposure bias in TRS.

Gunawardena and Sarathchandra (2020) suggest the use of deep neural networks to create a digital menu and personalize food item recommendations for customers, allowing them to make informed decisions. While the research does not specifically address fairness in recommendations, the approach could potentially be applied to the tourism industry as a means of providing fair food recommendations.

### 3.3. Platform fairness: ranking bias

Online platforms greatly impact offline experiences, such as selecting a tourist destination (Huang et al., 2018). The visibility of items on the platform is crucial to their success (Abdollahpouri and Mansoury, 2020). Items at the top of search results attract more attention, while those lower down may miss out on business opportunities (Craswell et al., 2008; Ursu, 2018). Additionally, platforms may be tempted to favor certain items more due to the commissions they receive from the item providers (Jannach and Bauer, 2020), which can lead to an unfair distribution of items on the platform and negatively impact some of its stakeholders. It's important for platforms to ensure fair item ranking to promote diversity in recommendations and ensure platform fairness. Unfair ranking can negatively impact stakeholders and erode trust in the platform. This paper analyzes the impact of unfair ranking on P-Fairness, focusing on platforms and item providers as individual stakeholders.

Apart from studies that have examined the impact of search rankings and position bias in different information retrieval scenarios [such as (Fortunato et al., 2006; Craswell et al., 2008; Chuklin et al., 2015; Baeza-Yates, 2018; Ursu, 2018; Geyik et al., 2019; Draws et al., 2021)], there have also been studies that have aimed to develop a fair ranking strategy specifically for the travel and tourism industry. TripAdvisor^8^, Airbnb, Booking.com, and Yelp are among the travel platforms that have been studied concerning the concept of fair ranking.

The authors of Li (2020) studied TripAdvisor data and found that Learning-to-Rank models based solely on implicit user feedback (such as clicks) can lead to bias. They proposed a method that takes into account the user's evaluation of all hotels above the clicked result and samples hotels below it based on their propensities. Their online experiment on TripAdvisor showed significant improvement in the search ranking using this method. Grbovic and Cheng (2018) propose search ranking methods tailored to Airbnb, using embedding techniques to personalize recommendations in real-time and effectively suggest home listings. Biega et al. (2018) introduced a notion of amortized fairness in ranking, which accumulates fairness over multiple rankings, resulting in improved individual fairness with high-ranking quality according to their study on Airbnb data. Gupta et al. (2021a) suggested re-ranking methods for online post-processing based on ranked batches of items, balancing fairness and utility, and performing well on Airbnb data. Lastly, Lahoti et al. (2019), focus on reconciling the fairness and utility of Airbnb data and propose a framework that results in individually fair learning-to-rank results. Mavridis et al. (2020) shed light on the multiple factors beyond the choice of algorithm that must be addressed for creating a machine-learned ranker in a large-scale commercial setting such as Booking.com. The authors suggest that their research could serve as guidance for applying machine learning to ranking problems. Another study by Kangas et al. (2021) address fair ranking in TRS platforms from a user experience perspective by developing a framework in Booking.com. This framework allows for the dynamic addition and removal of items, ensuring that new items have a fair chance, and enables recommendation blocks to be ranked in the most relevant order for the user interface. In their study, Zhu et al. (2020) propose a debiased ranking model that uses statistical parity and equal opportunity to mitigate item under-recommendation bias in personalized ranking systems. Their experiments on three publicly available datasets, including Yelp, demonstrate significant bias reduction compared to current state-of-the-art methods.

The concept of P-Fairness has also been explored for restaurant recommendations. For example, Kokkodis and Lappas (2020) proposed a fair ranking system for online platforms by examining the impact of the popularity-difference bias on online restaurant reviews. This bias stems from the difference in popularity between the reviewer's hometown and the destination being reviewed, which can lead to conflicting opinions on the effect of this bias on assigned ratings and review sentiment. The authors' analysis of a large set of restaurant reviews from a major online platform reveals a significant impact of this bias on restaurant ratings. They suggest that recognizing this bias can help online platforms improve their ranking systems, resulting in improved satisfaction for reviewers and more diverse recommendations for top restaurants.

Moreover, Gupta et al. (2022) present a novel solution to ensure fairness in food delivery services through the FairFoody algorithm. This algorithm uses delivery data to allocate fair income distribution among agents, while also ensuring timely deliveries. FairFoody's approach is unique in its focus on fairness in income distribution among agents, rather than just the recommendations. This could have potential applications in the tourism industry, such as fair allocation of resources among food vendors at a tourist destination.

To summarize, fair ranking in online platforms is essential in promoting diversity and building trust among customers and item providers. The online platforms have an ethical and moral responsibility to ensure that their recommendations are fair to all stakeholders. This fairness should be evident not only in terms of visibility and exposure but also in the ranking process to promote fair competition. Moreover, while there is a significant body of research focused on fair rankings in the context of hotel and restaurant recommendation platforms, there are few studies that address this concern for other tourism-related issues, such as trip planning or route optimization. Therefore, exploring fair ranking or ensuring P-Fairness in these areas presents a promising avenue for future research.

### 3.4. Societal fairness: sustainability

The impact of tourism extends beyond active participants to affect the local environment and businesses. Therefore, constructing a fair TRS requires considering sustainable recommendations. World Tourism Organization and United Nations Development Programme define *sustainable tourism* as “*tourism that takes full account of its current and future economic, social and environmental impacts, addressing the needs of visitors, the industry, the environment, and host communities*” (Gössling, 2017). Societal Fairness, also known as S-Fairness, focuses on meeting the needs of non-participating stakeholders in tourism, such as residents who may be affected by issues such as housing prices and congestion. In this context, we use the terms S-Fairness and Sustainability interchangeably.

Achieving sustainability in tourism requires various types of interventions, including municipal policies and regulations. To ensure sustainability in TRS, possible interventions include reducing the environmental impact of tourism, balancing the tourist load, promoting public transportation, encouraging carpooling, and supporting sustainable business practices. However, the idea of generating sustainable recommendations is a relatively new concept with limited literature available. Current literature on TRS focuses on regulating the number of tourists traveling to a destination to control the impact of tourism, particularly in preventing phenomena like over and undertourism.

The terms over and undertourism are used to describe situations where a destination is overwhelmed by too many tourists or lack tourists, respectively. Overtourism has become increasingly prevalent due to factors such as affordable transportation, home-sharing services, and exposure disparity caused by social media/recommendation technologies, leading to negative impacts on the environment, residents, and tourists' experiences (Camarda and Grassini, 2003; Rabanser and Ricci, 2005; Hospers, 2019; Dastgerdi and De Luca, 2023). Undertourism, on the other hand, occurs in lesser-known destinations with insufficient infrastructure, publicity, and accessibility, resulting in economic disadvantages (Gowreesunkar and Vo Thanh, 2020).

The idea of developing sustainability-driven recommender systems has recently received attention in the literature. For example, Merinov et al. (2022) have explained how recommender systems can potentially be used as a medium to introduce under-visited areas and strategically control tourists in over-visited areas through a case study on an Italian village. The authors proposed a multistakeholder utility model for travel itinerary optimization that protects popular destinations from overpopulating and less mature destinations from under-populating by distributing tourists throughout different points of interest (POIs) while preserving user satisfaction. The model used user preferences from the consumer side and time and occupancy of POIs from the environment side as two objectives and optimized the trade-off between the two using a greedy breadth-first search graph method to recommend optimal itinerary routes to users.

While research on the topic of over and undertourism in TRS is limited, the COVID-19 pandemic has sparked interest in utilizing RS to address these challenges and promote sustainable production systems. The pandemic has presented multiple challenges for businesses, including the need to maintain social distancing in public spaces such as restaurants and other venues. This has resulted in overcrowding in some places, compromising customer safety, and very low footfall in others, jeopardizing their economic sustainability. Patro et al. (2020b) addressed this issue by formulating it as a multi-objective optimization problem and mapping it to a bipartite matching with a polynomial time solution. Their experiments on real-world datasets from Yelp and Google Local^9^ have demonstrated that their model improves business sustainability, safety, and utility goals.

In addition, the pandemic has drawn attention to the importance of sustainable production methods in local businesses, with a focus on prioritizing the rights of local communities over the desires of tourists and the profits of tourism companies (Higgins-Desbiolles et al., 2019). To address this, Pachot et al. (2021) have proposed a novel recommender system for companies that takes into account territorial policies, while promoting diversity and providing a competitive advantage for providers. The objective of this system is not only to promote business growth, but also to consider factors such as economic growth, productive resilience, securing necessities, and sustainable production for local authorities. This approach offers a fresh perspective on the evaluation of S-Fairness in recommendations by emphasizing the involvement of local authorities (society), providing insights into an area of fairness in recommendations that have previously been unexplored.

To summarize TRS may have unintended consequences for other stakeholders who are indirectly involved in the process of recommendation. This highlights the importance of a holistic recommendation process that considers the perspectives and interests of all parties, including society. Initial research has indicated that TRS has the potential to effectively manage the allocation of limited resources, but its potential for addressing tourism-related concerns remains an open question.

## 4. Multistakeholder fairness in TRS

In certain applications, multiple fairness concerns may arise simultaneously for different stakeholders. Thus, a system may have any combination of the previously mentioned fairness considerations at play at once, such as for both consumers and providers, but also any other combination of stakeholders. Moreover, often the stakeholder concerns are conflicting, making it difficult to satisfy the specific concern of a single stakeholder. For example, a rental platform such as Airbnb and its rentals (item providers) share a common objective of avoiding position or popularity bias. To optimize the ranking of the rentals, it's necessary to simultaneously consider both P-Fairness and I-Fairness in this case. Therefore, in this section, we review methods that have simultaneously addressed more than one stakeholder in their fairness criteria.

To resolve the challenge of ensuring fairness toward multiple stakeholders, many studies adopt a multi-criteria optimization approach. This method involves optimizing a utility function that accounts for multiple criteria and preferences of various stakeholders while aiming to maintain a minimal trade-off in personalization. This approach is commonly used in other domains such as movies or music (Bouveret et al., 2016; Burke et al., 2016; Liu et al., 2019; Sühr et al., 2019; Patro et al., 2020a; Ranjbar Kermany et al., 2021), but has not yet been widely adopted in the field of tourism.

We have grouped the literature into three categories in Table 2: (1) works that specifically deal with fairness in TRS from multiple stakeholder perspectives, (2) works within the TRS domain that address multi-criteria recommendations, and (3) recent studies in other domains that address fairness in a multistakeholder scenario and can be adapted to the tourism industry.

**Table 2:** Summary of related works, and their main fairness criteria with an emphasis on relevant travel domain datasets (in bold).

			**Main fairness criteria**			
**Category**	**References**	**Dataset**	**C-fairness**	**I-fairness**	**P-fairness**	**S-fairness**
Fairness in TRS	Weydemann et al. (2019)	**Travel data solution**	•	•		
	Shen et al. (2021)	**Alibaba** travel marketing platform	•	•	•	
	Wu et al. (2021)	**Ctrip**, Google Local, and Amazon Review	•	•		
	Merinov et al. (2022)	synthetic data	•	°		°
	Rahmani et al. (2022b)	**Yelp**, **Gowalla**	•	•	•	
Multi-Criteria recommendations	Jannach et al. (2014)	**TripAdvisor**, HRS.com, YahooMovies	•	°		
	Nguyen et al. (2017)	**Expedia**	•	•	•	
	(Zheng, 2017a, 2019)	**TripAdvisor**, YahooMovies	•		°	
Multistakeholder fairness in other domains	Burke et al. (2022)	Kiva Microloans	•	•		
	Wu et al. (2022)	MovieLens	•	•		

The • signifies the acknowledged addressing of stakeholders, while the ° denotes a partial or incidental reference for each category.

### 4.1. Fairness in TRS

Fairness in TRS should be addressed from a multi-sided perspective owing to the involvement of multiple stakeholders in the system. In Section 3, the primary focus was on addressing fairness concerns for a single stakeholder. In this section, the focus shifts toward simultaneously optimizing fairness concerns for multiple stakeholders. The studies reviewed in this section use multi-objective optimization frameworks to generate fair recommendations in the tourism domain.

In the context of location-based recommendations Rahmani et al. (2022b) focus on addressing user fairness and item fairness for point of interest (POI) recommendations. They classify users into advantaged and disadvantaged levels based on their activity level and divide items into short-head, mid-tail, and long-tail groups to study their exposure in the recommendation list for users. They examine the interactions between different factors such as the unfairness of users (C-Fairness), the unfairness of popular items (I-Fairness), and the personalization offered by the recommender system (P-Fairness). Through evaluating their algorithms on publicly available datasets from Yelp and Gowalla, they found that most well-performing models suffer from popularity bias (provider unfairness). Furthermore, their study highlights that most recommendation models are unable to simultaneously satisfy both consumer and producer fairness, indicating a trade-off between these variables possibly due to natural data biases. Weydemann et al. (2019) explore the quantification of fairness in location recommendations. Their study focuses on different fairness aspects, and the results are based on data from Travel Data Solution, an Austrian company that equips rooms of certain hotels in Austria with cellular-based mobile hotspots. They evaluated different location recommenders against their defined fairness criteria and found that fairness depends on the specific fairness concerns being considered.

To mitigate the challenges of multi-scenario modeling and data fairness in the field of travel marketing, Shen et al. (2021) developed a model called the Scenario-Aware Ranking Network (SAR-Net). This model utilizes two specific attention modules to learn different scenarios by studying users' cross-scenario interactions. The proposed model was tested on Alibaba's travel marketing platform, resulting in a 5% increase in its clickthrough rate. They further suggest that this model can be applied to various travel scenarios to generate personalized and unbiased recommendations.

Wu et al. (2021) developed a two-sided Fairness-Aware Recommendation Model (TFROM) that utilizes post-processing heuristic algorithms to optimize for both C-Fairness and I-Fairness. The effectiveness of TFROM was evaluated using real-world flight data from Ctrip^10^, Google local dataset^11^, and Amazon review dataset^12^. The results of the experiments showed that TFROM provides better two-sided fairness while still having a minimal trade-off in personalization compared to the baseline algorithms.

Although multi-stakeholder utility models have been developed to address fairness criteria such as C-Fairness, I-Fairness, and P-Fairness, limited research has been conducted on S-Fairness, as shown in Table 2. A recent study by Merinov et al. (2022) has proposed a travel itinerary optimization approach to address S-Fairness by preventing overcrowding of tourist destinations. Their experiments were conducted on synthetic data and simulated scenarios, but further validations on real-life scenarios are required. This highlights the need for further research in this area to ensure fair recommendations for all stakeholders in actual tourism scenarios.

### 4.2. Multi-criteria recommendations

Several studies have been conducted on multi-objective optimization for recommendations on hotel booking platforms such as Expedia^13^ (Nguyen et al., 2017) and TripAdvisor (Jannach et al., 2014; Zheng, 2017a, 2019). Even though these studies are not explicitly concerned with fairness, they can be repurposed to generate fair recommendations.

For instance, Nguyen et al. (2017) propose a learning-to-re-rank approach for solving multi-objective recommendation problems involving multiple stakeholders. They demonstrate their solution in a detailed example using an in-house Expedia dataset, integrating multistakeholder issues in hotel recommendations by incorporating consumers, platform, and provider concerns. Similarly, Zheng (2019) use multi-criteria ratings from TripAdvisor and utilize the similarity or distance between expectation and rating vectors as the utility functions to map them to different aspects such as location, room size, and cleanliness, in the case of hotel booking. They use a scoring function to recommend top-N items to the user. Jannach et al. (2014) leverage customer feedback and satisfaction analysis from TripAdvisor data and improve recommendations. Another work "CriteriaChains" by Zheng (2017a) predicts utility values one by one in a chain-like structure. Their experimental evaluation based on TripAdvisor and YahooMovies^14^ rating datasets demonstrate that their proposed approach can improve the performance of multi-criteria item recommendations. The results of these studies show that these models provide improved two-sided fairness while maintaining a minimal trade-off in personalization.

### 4.3. Multistakeholder fairness in other domains

Outside the tourism domain, the topic of fairness in multistakeholder applications has received a lot of attention. While these systems differ from TRS, many of these fair RS approaches can be adapted to the tourism domain by redefining their fairness concerns.

Fairness in RS, including from a multistakeholder perspective, was surveyed by Deldjoo et al. (2022); Wang et al. (2022). The authors outline fairness definitions in recommendations and classify fairness issues from various perspectives. They also summarize the datasets and measurements used in fairness studies and present a comprehensive taxonomy of fairness methods in recommendations. We refer to their papers for an in-depth review. In this paper, we discuss additional recent studies which have not been addressed in the aforementioned work.

In the work by Wu et al. (2022), the authors propose a multi-objective optimization framework called Multi-FR for addressing the issue of multistakeholder fairness-aware recommendation. Multi-FR jointly optimizes for accuracy and fairness for both consumers and producers in an end-to-end way, resulting in a guaranteed Pareto optimal solution. The authors evaluated their model using the MovieLens dataset, but the approach can be adapted for other domains such as tourism. Another related study is the work of Burke et al. (2022), in which they introduce an innovative architecture for implementing multistakeholder fairness in recommendation systems, where fairness concerns are represented as agents in a dynamic social choice environment. They evaluated their approach on Kiva Microloans,^15^ an online loan lending platform, and show that it outperforms baseline methods. Similar to the domain of tourism, where the needs of different stakeholders need to be balanced, this approach can be adopted while redefining fairness concerns.

Although multi-objective optimization appears to be a promising approach for ensuring fairness for all stakeholders, it often involves a trade-off with other criteria, such as reduced user satisfaction. This outcome is counterproductive as the primary objective of a recommender system is to recommend items that fulfill user needs. Additionally, the metrics used to measure fairness are highly dependent on the domain and context and require adaptation. Moreover, most studies evaluate their models through offline analysis using either existing or synthetic datasets. Unfortunately, they lack emphasis on user acceptance of the re-ranked or fairly recommended results. Furthermore, the use of synthetic data may not accurately reflect real-life scenarios, particularly in a dynamic domain such as travel and tourism. Consequently, future research must address these issues.

## 5. Challenges in fair recommender systems in tourism

Tourism is a highly dynamic and rapidly growing industry, and recommender systems have become an essential tool in helping tourists make informed decisions. However, the implementation of fair and equitable recommender systems in the tourism industry presents numerous challenges. The complexity of balancing the needs and preferences of multiple stakeholders, such as tourists, service providers, and platform providers, creates a complex decision-making environment. Additionally, factors such as changing contexts and the diversity of domains add to the complexity of the problem.

In this section, we will examine the challenges associated with designing fair and balanced tourism recommender systems and explore possible solutions for mitigating these challenges. Through our examination of existing literature, we have identified these challenges, which can serve as valuable areas of focus for future research into developing fair tourism recommender systems. The section is organized as follows: we begin by examining the challenges faced by individual stakeholders in Section 5.1, then consider the trade-offs between different stakeholder groups in Section 5.2, explore how explanations can enhance user interfaces and transparency in Section 5.3, and conclude by addressing the shortage of publicly available data, metrics, and evaluation approaches in Section 5.

### 5.1. Modeling individual stakeholder utilities

In the tourism industry, modeling utilities for each stakeholder is essential, similar to other recommendation domains. However, utility modeling in tourism is a complicated process, as it is often influenced by dynamic factors such as context, seasonality, travel regulations, etc. The work by Zheng (2017b) effectively illustrates the difficulties encountered in a multistakeholder travel recommendation scenario. The authors emphasize the importance of considering the correlation among utilities due to dynamic factors that can impact stakeholder preferences. They note that these preferences may vary and be influenced by changing circumstances such as contextual factors or emotional states. For instance, when making a multi-criteria hotel booking recommendation on TripAdvisor.com, room size may be a crucial factor for a user when planning a family trip. A low rating on room size can directly influence the user's rating on other criteria such as “value” and overall rating of the hotel. To include these correlations in the model, researchers like Sahoo et al. (2012) have developed probabilistic recommendation algorithms based on pre-defined graphical relationships. Another proposed approach, “CriteriaChains” (Zheng, 2017a), predicts utility values one by one in a chain-like structure.

Our research has revealed that, although there has been some exploration of modeling the utilities of individual stakeholders such as consumers, providers, and platforms, there has been limited attention paid to the role that society plays in the recommendation process. In particular, the concept of sustainable tourism has been largely overlooked in recommendations, despite its importance in balancing the challenges of over and undertourism and reducing the environmental impact of tourism activities. A potential solution to combat over and undertourism could be optimizing the crowdedness of a location. Although there is some information available on Google, it is not readily accessible. Improving crowdedness modeling and increasing information on this topic will not only help mitigate overtourism but also support sustainable tourism. This highlights the need for a more comprehensive approach to the recommendation process in the tourism industry, one that takes into account the interests of all stakeholders, including society.

### 5.2. Complexity in inter-stakeholder relationships

Recommending items that meet the needs of multiple stakeholders is a challenging task, as it requires balancing different preferences and goals. Although research in this area has been conducted, there are relatively few studies specifically focused on TRS. Some approaches address the problem as an optimization problem (Weydemann et al., 2019; Shen et al., 2021; Wu et al., 2021), while others focus on providing transparent explanations for recommendations (Wang et al., 2022). However, the complexity of the problem is further compounded by factors such as shifting contexts and the diversity of domains (explained in subsection 5.1). As a result, solutions for multistakeholder TRS often involve making trade-offs among various optimization parameters (Rahmani et al., 2022b).

The relationships between stakeholders can be intricate and can impact their interactions and outcomes. In the tourism industry, for example, a consumer's relationship with the item provider can influence their ratings positively or negatively, leading to bias and unfairness in TRS Zheng (2017b). In Balakrishnan and Wörndl (2021), the authors highlight how one stakeholder's gain can negatively impact others in the same group, particularly in tourism recommender systems. For example, a user receiving a discount on a resort could cause another tourist to miss out, and a provider being selected by a customer could result in a loss of utility for other providers. To address this, the authors suggest using value-aware recommender systems that take into account both user and stakeholder utility gains (Pei et al., 2019; Abdollahpouri et al., 2020). As such, designing these systems in the multistakeholder context is a good starting point for a fair TRS that balances stakeholder utilities.

Temporal factors can also affect the relevance of recommendations. While context-aware recommendation models (Zheng et al., 2014, 2016) may improve the quality of recommendations, it is important to evaluate the effectiveness of multistakeholder recommendations in different contextual situations. Moreover, travel restrictions and tourism trends play an integral role in the design and implementation of TRS, as noted by Balakrishnan and Wörndl (2021). The authors emphasize that taking into account the impact of external factors can greatly enhance the benefits of a multistakeholder RS, including enhanced user satisfaction, higher conversion rates, and increased provider exposure. They categorize external influences into four groups based on duration and predictability: constant, deterministic recurrent, non-deterministic recurrent, and volatile. However, these external influences can pose significant challenges in designing fair TRS, as they are difficult to quantify and incorporate into recommendations.

### 5.3. Explanations to improve user interfaces

Fairness in recommendation is essential in ensuring that the recommendations generated do not favor any particular individual or group of individuals, such as consumers or providers. This can be achieved through both the fair usage of information in the recommendation process, as well as by ensuring that the recommendations themselves are fair (Zhang et al., 2020). Additionally, providing explanations or reasoning behind the recommendations can help users understand the fairness objectives of the recommender system, and potentially impact their perceptions of the fairness of the system. Explanations can also provide transparency, increase efficiency, effectiveness, and trust in the system, and ultimately lead to increased user satisfaction (Tintarev and Masthoff, 2010).

Explainability in recommender systems allows for the justification of a model's predictions and the identification of potential biases. It is an effective tool for increasing fairness in various branches of AI (Sonboli et al., 2021). Many researchers have also explored the relationship between explainability and fairness in recommender systems. For example, Abdollahi and Nasraoui (2018) argue that traditional metrics such as accuracy do not account for fairness in recommendations, and thus, explainable models are needed to achieve fairness. Similarly, Sonboli et al. (2021) suggest that it is not enough to simply claim a system is fair, rather, fairness goals should be effectively explained to users for them to perceive the recommender system as fair. Another study by Elahi et al. (2021) uses the Universal Design for Learning (UDL) framework to introduce three metrics for evaluating user-perceived fairness in recommender systems: Engagement, Representation, and Action & Expression; and suggests that explanations can contribute to fairness in the representation of recommendations.

By offering explanations for recommendations, transparency, trust, and user satisfaction can be promoted, and users can make more informed decisions. Despite the importance of this issue, research on how to explain recommendations with a multistakeholder fairness objective in the tourism industry is limited.

### 5.4. Insufficient data, missing metrics, and evaluation

The study of fairness in TRS is an emerging field, but a lack of publicly available data hinders its progress. Many studies in this area have used synthetic datasets (Merinov et al., 2022) or data from specific platforms that are not publicly accessible. For example, some have used an in-house dataset from Expedia, which is not available to the general public (Nguyen et al., 2017). Additionally, the available datasets often lack essential information such as user interactions or preferences and typically contain only limited fairness-related metadata such as gender and age. Moreover, to address environmental impact and incorporate societal perspectives into the recommendation process, it is essential to have access to data that quantifies metrics such as environmental impact and the crowdedness of a place. This makes it difficult to reproduce results or generalize findings. The data availability problem is also present in the music domain, as noted by Dinnissen and Bauer (2022). Although there is debate on whether such data should be made publicly available, it is clear that more representative and detailed data is needed to develop effective and fair TRS.

The fairness metrics used in fair TRS research are highly specific to a particular domain or context, making it challenging to generalize their application. The complexity of modeling utilities in the tourism domain, as discussed in Section 5.1, further complicates the issue. Despite the advances made by recent methods like Bauer et al. (2023), which provide researchers with tools for carrying out, analyzing, and comprehending recommendation computations through the use of 5 datasets, 11 metrics, and 21 recommendation algorithms, it is not possible to extend its results to all scenarios.

Furthermore, achieving fairness in recommendation systems is a complex task due to the societal construct of fairness, with various definitions existing (Narayanan, 2018). To address this, several researchers have proposed different ways of operationalizing fairness constraints. However, many of these approaches lack evidence or argumentation justifying the chosen fairness metrics' practical relevance in general or specific application settings (Jannach and Abdollahpouri, 2023). Some prior works, including Abdollahpouri et al. (2019a), have loosely associated fair recommendations with reducing popularity bias by matching with a target distribution or metric threshold. However, as pointed out by Jannach and Abdollahpouri (2023) it remains unclear what normative claim justifies recommending less popular items, which could be of poor quality and perceived as unfair by users. Moreover, recommending mostly popular items may negatively impact accuracy and affect different user groups in distinct ways, as shown in previous studies on movies (Abdollahpouri et al., 2019b) and music (Kowald et al., 2020) domains.

While most studies evaluate their models through offline analysis or using existing datasets, there is a lack of focus on user acceptance of the re-ranked or fair recommended results. This is a vital aspect of recommender systems, as they must not only align with user preferences but also be fair to all stakeholders. Future research should prioritize this aspect to ensure the practicality and effectiveness of the models developed.

## 6. Conclusion

In recent years, the prevalence of unfairness in recommender systems has become a topic of increasing concern, leading to the development of various definitions, metrics, and techniques to promote fairness. As a multi-faceted concept, ensuring fairness in recommender systems involves addressing the needs of multiple stakeholders, both within and beyond the system. This paper reviews existing literature on fairness in tourism recommender systems, categorizes stakeholders based on their primary fairness criteria and discusses the challenges associated with developing fair recommender systems.

While research has been done on fairness in RS in other domains, the domain of travel and tourism remains largely unexplored. The majority of studies in the tourism sector have centered on fairness in accommodation and restaurant recommendations, while other areas such as fair trip planning and transportation have received limited attention. Additionally, there has been limited research into integrating societal concerns as a stakeholder when defining the utility function of a recommendation. Future work should prioritize balancing the requirements of society with those of the other stakeholders.

## Author contributions

AB: literature analysis. PB: literature analysis, contributed to Sections 3 and 4. WW: supervised the project. All authors contributed to the manuscript revision, read, and approved the submitted version.

## Footnotes

^1^ https://www.airbnb.com/

^2^ https://www.booking.com/

^3^ http://snap.stanford.edu/data/loc-gowalla.html

^4^ https://www.yelp.com/dataset

^5^ https://www.hrs.com/

^6^ https://www.last.fm/

^7^ https://grouplens.org/datasets/movielens/

^8^ https://www.tripadvisor.com/

^9^ https://developers.google.com/maps/documentation/places/web-service/overview

^10^ https://www.ctrip.com

^11^ http://jmcauley.ucsd.edu/data/googlelocal/

^12^ https://jmcauley.ucsd.edu/data/amazon/

^13^ https://www.expedia.com/

^14^ https://www.yahoo.com/entertainment/movies/

^15^ https://www.kiva.org/
